# Measurement of Cyanobacterial Bloom Magnitude using Satellite Remote Sensing

**DOI:** 10.1038/s41598-019-54453-y

**Published:** 2019-12-04

**Authors:** Sachidananda Mishra, Richard P. Stumpf, Blake A. Schaeffer, P. Jeremy Werdell, Keith A. Loftin, Andrew Meredith

**Affiliations:** 1 Consolidated Safety Services Inc., Fairfax, VA 22030 USA; 2 National Oceanic and Atmospheric Administration, National Centers for Coastal Ocean Science, Silver Spring, MD 20910 USA; 3 Office of Research and Development, U.S. Environmental Protection Agency, Durham, NC 27709 USA; 4 Ocean Ecology Laboratory, NASA Goddard Space Flight Center, Greenbelt, MD 20771 USA; 5 U.S. Geological Survey, Organic Chemistry Research Laboratory, Kansas Water Science Center, Lawrence, KS 66049 USA

**Keywords:** Limnology, Environmental sciences

## Abstract

Cyanobacterial harmful algal blooms (cyanoHABs) are a serious environmental, water quality and public health issue worldwide because of their ability to form dense biomass and produce toxins. Models and algorithms have been developed to detect and quantify cyanoHABs biomass using remotely sensed data but not for quantifying bloom magnitude, information that would guide water quality management decisions. We propose a method to quantify seasonal and annual cyanoHAB magnitude in lakes and reservoirs. The magnitude is the spatiotemporal mean of weekly or biweekly maximum cyanobacteria biomass for the season or year. CyanoHAB biomass is quantified using a standard reflectance spectral shape-based algorithm that uses data from Medium Resolution Imaging Spectrometer (MERIS). We demonstrate the method to quantify annual and seasonal cyanoHAB magnitude in Florida and Ohio (USA) respectively during 2003–2011 and rank the lakes based on median magnitude over the study period. The new method can be applied to Sentinel-3 Ocean Land Color Imager (OLCI) data for assessment of cyanoHABs and the change over time, even with issues such as variable data acquisition frequency or sensor calibration uncertainties between satellites. CyanoHAB magnitude can support monitoring and management decision-making for recreational and drinking water sources.

## Introduction

Cyanobacterial harmful algal blooms (cyanoHABs) are a serious environmental, water quality and public health issue worldwide because of their ability to form dense biomass and scum and to produce toxins such as neurotoxins (anatoxin-a), hepatotoxins (microcystins), and cytotoxins (cylindrospermospin)^1^. Cyanotoxins are capable of causing a wide variety of adverse human health issues including gastrointestinal distress, dermatitis, liver failure, or even death of domestic and livestock animals when they are exposed to water with toxins from intense cyanoHABs^2,3^. CyanoHABs are considered to be increasing globally over the past few decades although observations are quite limited^4,5^. The frequency and magnitude of these blooms are expected to worsen in the future with increased surface water temperatures and vertical stratification^6^. In addition, cyanoHAB growth and intensity are known to be affected by weather-driven environmental and anthropogenic factors, such as shifts in rainfall patterns with climatology and changing agricultural practices^7–9^.

In order to reduce the risk of exposure to cyanotoxins, more frequent water quality assessments are needed to monitor the status and historical trends of cyanoHABs in inland lakes. This information is needed at regional as well as national scales, including lakes and reservoirs designated as drinking water sources and recreational water bodies. In the United States, the National Lakes Assessment (NLA)^10^ was designed to provide national estimates of lake conditions with biological, chemical, physical, and recreational/human health indicators. Through this program, assessments of ecologically representative samples of U.S. lakes >1 ha in size are conducted every five years and were last completed in 2012 (2017 is pending). However, a yearly assessment of individual lakes will aid in developing management strategies.

Lake assessments using traditional field sampling methods (routine laboratory analysis for measurements of phytoplankton pigment and cell concentration, biovolume, and biomass) are expensive, time-consuming, and often not feasible to carry out in multiple waterbodies or across multiple states. However, satellite-based remote sensing methods can be used to monitor the current status of cyanoHABs in numerous larger water bodies on a routine basis^11,12^ and to retrospectively assess the historical status of these water bodies^13^. Among cyanoHAB assessment studies using remote sensing methods, a few have looked at several lakes at a time^14–16^, but a majority of the efforts have focused on one body of water such as the Baltic Sea^17^, Lake Balaton, Hungary^18^, the Caspian Sea^19^, Lake Taihu, China^20,21^, and Lake Erie^11,12,22^.

Researchers often quantify cyanobacteria as biomass measured directly or by surrogate approaches such as with concentrations of chlorophyll-a (Chl-*a*) (mg m^−3^)^17,22–24^, phycocyanin (PC) (mg m^−3^)^25–27^, or as cell concentrations^12,28^. Recent studies on multiple lakes have focused on quantifying cyanoHAB spatial extent (km^2^)^29^ and temporal frequency (% of observations)^15^. These studies have started to address management questions of change in spatial and temporal cyanobacteria bloom dynamics over time. Resource managers have limited resources for assessment and monitoring of lakes for public and environmental health. Knowing which lakes have severe or worsening blooms, and which do not, allows the managers to determine viable lake management strategies. An indicator of bloom magnitude would provide a key addition to the previous metrics by characterizing algal bloom biomass for an observational time (season or year), thereby highlighting the annual scale of the blooms. In this study, we introduce a metric that focuses on the magnitude of cyanoHABs in lakes and other inland water bodies. We define bloom magnitude as the spatiotemporal mean of the sequence of 7 or 14-day composites of maximum bloom biomass collected over the bloom season. In order to make the bloom magnitude comparable across lakes of different size, magnitude is further normalized to lake surface area. We also use rank as a key metric to compensate for significant changes in data frequency, and ultimately other factors like differences between satellites or calibration drift.

Nine years of European Space Agency (ESA) MEdium Resolution Imaging Spectrometer (MERIS) data (2003–2011) with a nominal pixel resolution of 300 × 300 m was used to estimate the annual magnitude of cyanoHABs in Florida and Ohio lakes. The primary objectives of this study were: (1) to develop a method for estimating annual cyanoHAB magnitude in inland lakes and reservoirs using MERIS observations; and (2) to generate a baseline cyanoHAB magnitude dataset during the 2003–2011 time-period in Florida and Ohio as a case study. The methods are applicable to the Sentinel-3 Ocean Land Color Imager (OLCI), the replacement for MERIS, which was first launched on Sentinel-3A in 2016.

## Data and Methods

### Study area

**Figure 1:**
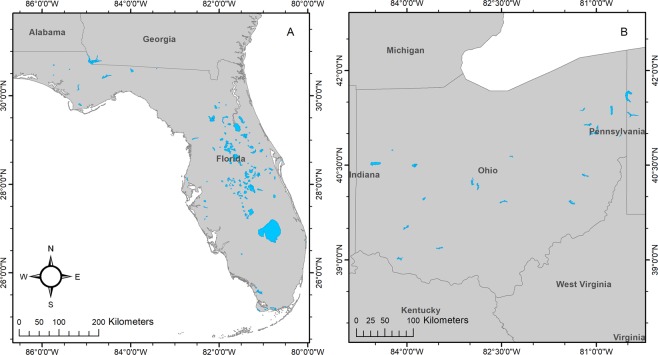
Map of the study region showing the location of lakes in (**A**) Florida and (**B**) Ohio. In total, 135 lakes in Florida and 21 lakes in Ohio, were resolvable with the full resolution MERIS data and are used in this study. Land and lakes are shown in gray and blue colors respectively.

We selected the states of Florida and Ohio as our study areas for three primary reasons (Fig. 1):

1. **To examine cyanoHAB magnitudes in lakes that are known to have cyanoHAB related water quality issues**. These two states also have a significant number of lakes that are resolvable in MERIS/OLCI data. Many lakes in the Coastal Plains ecoregion, which includes Florida, are known to have cyanoHAB issues, with 34% of lakes known to be hypereutrophic by the NLA in 2007^30^. The 2007 NLA also reported that 43% of lakes in Florida had microcystin present^31^. Similarly, cyanoHABs are a common water quality issue in the Temperate Plains ecoregion, which includes western Ohio, where 45% lakes are considered hypereutrophic^30^. Approximately 32% of lakes in Ohio had microcystin present in 2007^31^.
2. **To consider results from lakes located in different geographic and climatic regimes**. The climate in Florida is subtropical, with hot, humid, high precipitation summers and mild, dry winters. In contrast, Ohio has a temperate climate with cold winters, hot and humid summers, and year-round moderate precipitation^32^.
3. **To assess the impacts of differences in data coverage in each location**. MERIS full resolution (FR) data collection frequency prior to 2008 was inconsistent. The temporal frequency of MERIS FR data over Ohio is higher than that over Florida during this time period. The consideration of two states with different temporal data coverage will illustrate the effect of reduced data frequency on the bloom magnitude metrics.

**Figure 2:**
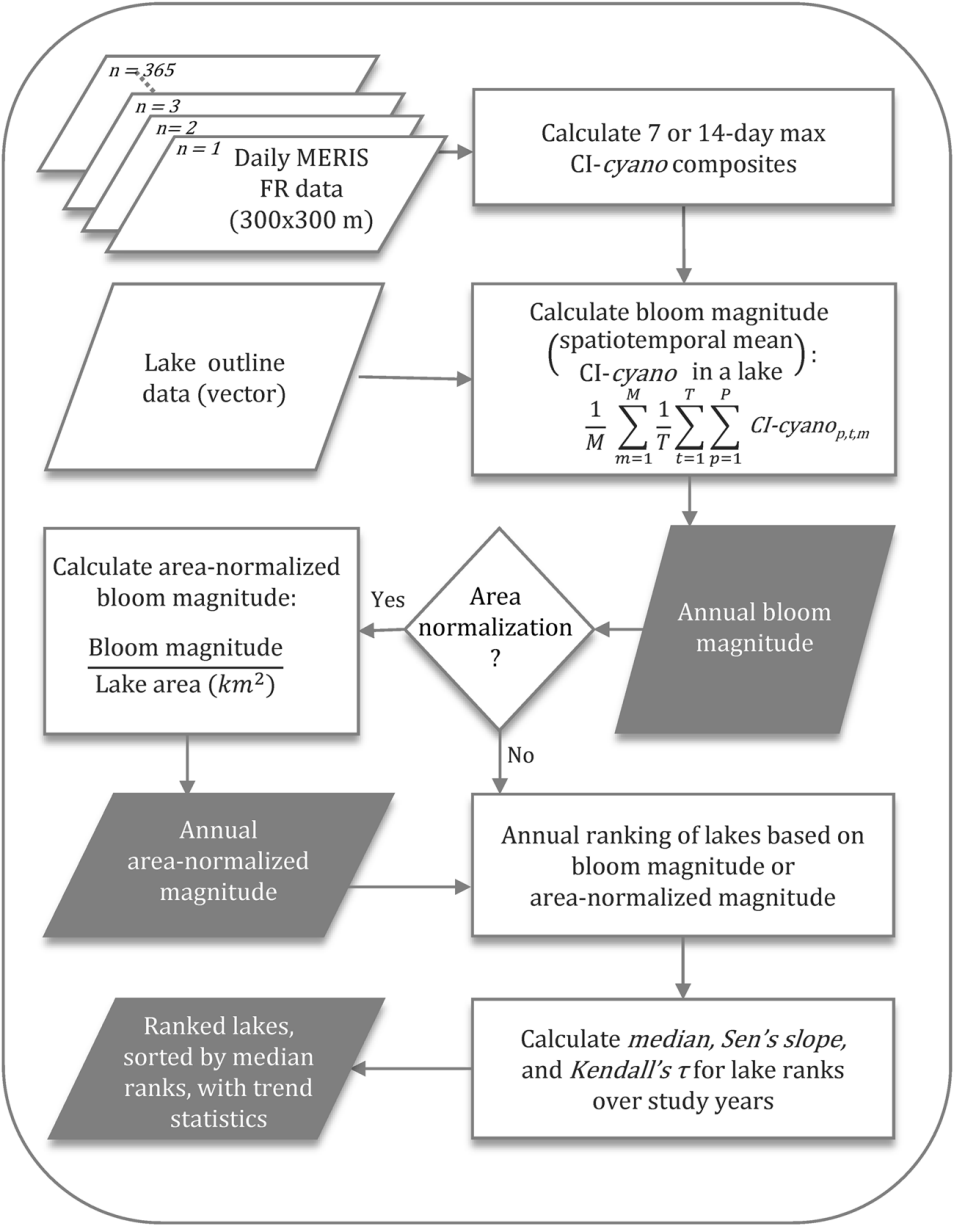
Schematic diagram of data processing and workflow for calculating bloom magnitude and area-normalized magnitude. Output stages in the workflow are shaded gray.

Figure 2 shows the steps of the data analysis and workflow carried out in this study. Individual components of the data and methods are presented below.

### Lake outline data

The lakes were screened for size using polygons of lakes and water bodies from the National Hydrography Dataset Plus version 2.0 (NHDPlusV2) lake polygons dataset^33^, with the condition that each selected water body should be resolved by a satellite image with 300 × 300 m pixel resolution. Lakes and other water bodies were considered resolvable if they had the equivalent of three connected non-mixed water pixels (i.e. three pixels without any land) within the NHDPlusV2 dataset. Further, all selected water bodies were screened and filtered using U.S. EPA’s 2012 NLA^34^ site evaluation guidelines (U.S. EPA, 2011). Waterbodies classified as intermittent or estuarine were excluded from further analysis based on NLA criteria (although some estuarine lakes in Florida were not identified and excluded, as discussed later). The final lake polygon layer included 135 lakes in Florida and 21 lakes in Ohio that would be resolved in FR MERIS/OLCI imagery. The surface area of resolvable lakes in Florida varied from 1.26 km^2^ to 1427 km^2^ with a median surface area of 5.31 km^2^, whereas, surface areas of Ohio lakes varied from 1.98 to 53 km^2^ with a median of 8.9 km^2^. In Florida, Center Lake and Lake Okeechobee were the smallest and largest lakes considered in this study, respectively. In Ohio, Evans Lake and Pymatuning Reservoir were the smallest and largest, respectively.

### Satellite data

MERIS Level-2 (L2) datasets were processed using the satellite automated processing system (SAPS) administered by the National Oceanic and Atmospheric Administration (NOAA). SAPS incorporates l2gen, the NASA standard software for processing L2 ocean color data that is included in NASA’s SeaWiFS Data Analysis Software (SeaDAS) package^35^, and the Shuttle Radar Topography Mission (SRTM) global land mask^36^. The l2gen-derived “rho_s” surface reflectance (*ρ*_*s*_ (unitless)) product was projected to Universal Transverse Mercator (UTM) projection using nearest-neighbor interpolation. The rho_s process generates a reflectance that corrects for top-of-atmosphere solar irradiance and removes Rayleigh radiance and molecular absorption corrected for elevation. Clouds are masked using an albedo threshold algorithm corrected for turbid water, with the latter necessary to retain those pixels with bright reflectance from intense blooms that would otherwise be incorrectly masked as clouds. Land adjacency issues, including mixed land/water pixels, were detected using near-infrared and red-edge thresholds^37^, thereby ensuring that the signals originating from land vegetation were flagged and excluded from further analysis.

Temporal coverage and data density for the MERIS FR data has changed over the mission. Prior to 2008, MERIS FR data sets for North America were obtained by onboard recording, which limited data acquisition. From 2002–2004, there was also competition for band width with other ENVISAT instruments, which may have also reduced acquisition frequency. In 2008, the Canadian Centre for Remote Sensing began reception of direct broadcast, which assured acquisition of most North American data^38^. In the contiguous United States (CONUS), prior to 2008, FR data frequency was lower over the southern states, including Florida^39^. Our analysis showed that the number of composites with near-100% missing data (inclusive of clouds and no satellite data collected) varied significantly in Florida and Ohio. The mean percentage of composites with near-100% missing data varied from 13% to 64% in Florida from 2003–2007. During the same time period, the mean percentage in Ohio varied from 12–28%. In contrast, from 2008–2011, the percentage of composites with missing data decreased to 6–12% (in Florida) and stayed about the same (14–29%) in Ohio. When the study period was restricted to 2008–2011 recreational months (May 1^st^ to Oct 31^st^ as per Ohio Environmental Protection Agency (Ohio EPA) recreational criteria^40^), Florida lakes received slightly more data coverage as compared to Ohio lakes as shown by the means of missing data percentage in Florida (range = 1–9%) and Ohio (0–16%).

### Cyanobacteria estimation algorithm

Cyanobacterial quantity was found through a combination of biomass estimation and cyanobacterial presence algorithms^11,28^. The Cyanobacteria Index (CI) measures a proxy of Chl-*a* absorption and provides the cyanobacterial biomass^11,18,19,29,41^. It is calculated with a spectral shape (SS) algorithm^28,42^ and is presented as:

1 $$SS(\lambda )={\rho }_{s}(\lambda )-{\rho }_{s}({\lambda }_{-})+\{{\rho }_{s}({\lambda }_{-})-{\rho }_{s}({\lambda }_{+})\}\frac{(\lambda -{\lambda }_{-})}{({\lambda }_{+}-{\lambda }_{-})}$$

where *ρ*_*s*_ is the top of atmosphere reflectance corrected for Rayleigh radiance, λ is the central band, and λ_+_ and λ_-_ are the adjacent reference bands. Cyanobacteria Index (CI) is calculated by centering the spectral shape at 681 nm and changing the sign of SS, or CI = −SS (681).

The CI evaluates Chl-*a* absorption at 681 nm. At 681 nm, chlorophyll in eukaryotes typically fluoresces strongly, leading to increased apparent reflectance that obscures chlorophyll absorption. In cyanobacteria, however, chlorophyll fluorescence is much weaker^43^, such that Chl-*a* absorption dominates the radiance signal from the water at 681 nm, causing the reflectance at 681 nm to decrease relative to 665 and 709 nm.

For more specific identification of cyanobacteria, a SS using 620, 665, and 681 nm was used to identify the presence of PC, a characteristic pigment in this taxonomic division with identifying features in this spectral region^25,41,44^. (Estimated PC concentration is not used as it has several issues, in particular, it is not a consistent estimator of cyanobacterial biomass, and the more usable PC concentration algorithms require robust atmospheric corrections^45^ greatly limiting data availability.) In this case, a conditional negative SS (665) value indicates the presence of PC. Inclusion of 620 nm, which is the absorption peak of PC, a characteristic photopigment in cyanobacteria, reduces the false detection issue. In the case of cyanobacteria, SS (665) turns negative due to lower reflectance at 620 nm band and is used as an exclusion criterion to select only cyanobacteria. This spectral shape condition has also been applied by^46^ (their Eq. 3–4) to separate cyanobacteria from other algal blooms in African lakes. The CI product, when SS (665) is negative, is termed as CI-*cyano* and was used to estimate cyanobacteria biomass in this research.

For purposes of setting risk thresholds, we applied a relationship between CI and cyanobacterial cell concentration of 10^8^ cells mL^−1^ per 1 unit of CI-*cyano*^11^. While the relationship^11^ was developed for *Microcystis* (so we term the value as “*Microcystis*-equivalent cells”), it was validated by^15,41^ for unspecified total cell concentrations of cyanobacteria in eight U.S. eastern states across New England (Connecticut, Massachusetts, Maine, New Hampshire, Rhode Island, and Vermont), Ohio, and Florida. Mean absolute percent error (MAPE) of 28.6% was reported between field-measured cyanobacteria biomass data (cells mL^−1^) and satellite-derived cell biomass^15^. This CI algorithm has also been confirmed for detecting cyanobacterial blooms and estimating biomass (cells mL^−1^) in other areas^18,19^.

Fourteen-day and seven-day maximum temporal CI-*cyano* composites were computed for 2003–2007 and 2008–2011 MERIS FR time series data. Fourteen-day intervals were chosen for compositing the 2003–2007 time series in order to address the FR data gaps as discussed earlier. Maximum temporal composites, that is, reporting of the maximum value retrieved during the 14-day or 7-day window, serve two purposes. First, many cyanoHAB species such as *Microcystis*, *Aphanizomenon*, and *Dolichospermum*, have buoyancy control mechanisms and will typically float to the surface in the day when vertical water column mixing is weak^47^. It is expected that over a 14-day or 7-day window, cyanobacteria would be near the surface on one or more days to be captured in the satellite data^12,47^. In addition, compositing reduces the amount of missing data, particularly due to clouds and sun-glint. The set of 14-day composites helped to reduce the under-sampling bias before 2008 (that was caused by reduced frequency in FR data acquisition discussed earlier).

### Annual or seasonal bloom magnitude metric

CyanoHAB magnitude is intended to capture the combination of two key aspects of an algal bloom: the amount and duration of the cyanoHAB biomass. The annual/seasonal bloom magnitude is the mean biomass of the 14-day or 7-day maxima found in the lake over a year/season and mathematically expressed as:

2 $$Bloom\,magnitude=\frac{1}{M}\,\mathop{\sum }\limits_{m=1}^{M}\frac{1}{T}\mathop{\sum }\limits_{t=1}^{T}\mathop{\sum }\limits_{p=1}^{P}{\mathrm{CI}-\mathrm{cyano}}_{p,t,m}$$

where, the indices *P* and *T* represent, respectively, the number of valid pixels with detectable CI-cyano in a lake, and the number of composite (time) sequences in each month (e.g. two composites in 2003 and four in 2011). Index *M* represents the number of months in a season/annual study period. Bloom magnitude was expressed in CI units, which is dimensionless. As noted above, CI-*cyano* can be converted to *Microcystis-equivalent* cells by multiplying by the factor 10^8^ (cells mL^−1^)^11,15,41^, to provide a more intuitive biomass metric. In order to be able to compare bloom magnitude across lakes with different surface area, we normalize bloom magnitude by lake surface area as below:

3 $$\mathrm{Area}-\mathrm{normalized}\,{\rm{bloom}}\,{\rm{magnitude}}=\frac{Bloom\,magnitude\,(dl)}{Lake\,Surface\,Area\,(k{m}^{2})}$$

Lake surface area in Eq. (3), as detectable by satellite images, was estimated by identifying all pixels inside a lake polygon vector layer that were classified as water during 2008–2011. The number of water pixels was converted to area by multiplying it by area of one MERIS FR pixel (0.09 km^2^). Note that the estimated surface area may change over time due to seasonal precipitation and evapotranspiration. However, this satellite-adjusted surface area is a better representation of surface area than that available in lake databases, which often include dry lake beds and/or areas not covered by standing water. Henceforth, bloom metrics in Eqs. 2 and 3 are referred to as magnitude and area-normalized magnitude for brevity.

Based on the World Health Organization’s (WHO) cell abundance threshold^48^, three magnitude classes were considered for categorizing lakes as Low (≤20,000 cells mL^−1^), Moderate (20,000 ≤ cells mL^−1^ ≤ 100,000), and High (>100,000 cells mL^−1^) exposure health risk. We estimated the area-normalized magnitudes that are equivalent to the WHO thresholds using the CI-cell abundance relation^11,15,41^ and normalizing this cell-equivalent threshold by the pixel unit area (CI threshold/0.09 km^2^). These area-normalized CI thresholds are ≤0.0022 for WHO-low, 0.0022 to 0.0111 for moderate, and >0.0111 for high. We have also added ‘Very High’ (V.High) category when estimated cyanobacteria concentration and area-normalized magnitude exceeded 1,000,000 cell mL^−1^ and 0.111, respectively.

### Ranking of lakes based on the area-normalized magnitude

Satellite data frequency from 2003–2007 was not spatially homogeneous across CONUS as discussed earlier. Varying MERIS FR data frequency in the CONUS adds a bias in the MERIS time series data which, in turn, can bias comparisons of lakes across space and time. As a result, the analysis of trends in the area-normalized magnitude data across 2003–2011 could produce misleading results^15,29^. To address this issue, the lakes were ranked based on their seasonal or annual area-normalized magnitude with rank 1 assigned to the lake with the greatest area-normalized magnitude in a specific year. When more than one lake had the same area-normalized magnitude level, the minimum possible rank was assigned to all lakes in the group. To summarize across years, each lake’s median rank for the observational period was used. While ranks reduce the information on absolute bloom impact that is found in magnitude, they offer a key advantage over magnitude by allowing us to compare lakes between years, even when differences in data frequency biases the magnitudes. Ranks might still be region-specific, depending on differences in data coverage. For example, magnitude scores cannot be compared (ranked) between regions (states) having widely varying data sampling, because the lakes with sufficiently higher data frequency might appear to have more severe blooms than the lakes with reduced frequency. Lakes in regions (states) with similar data coverage can be compared with minimal bias.

We used a non-parametric statistic, Theil-Sen’s slope estimator^49^ for assessing trends in the ranks of cyanoHAB magnitude in a lake over time, with Kendall’s *τ*^50^ for the strength of the trend. Theil-Sen’s slope is estimated as the median of the set of slopes in the ranked and paired data. Theil-Sen’s estimator for slope makes no assumptions about error distribution and provides an unbiased estimator of trend^51^. Theil-Sen’s slope was expressed in the units of ranks yr^−1^ and interpreted as the number of ranks increased or decreased over time for a lake in question. A negative trend (toward the rank of 1) indicates that the lake is getting relatively worse. Kendall’s *τ*, by determining the concordance between all pairs of two ranked variables indicates the strength of a monotonic trend^51^. Kendall’s *τ* varies between −1 and +1, where a positive *τ* indicates that the ranks of both variables increase together, and a negative *τ* indicates that as the rank of one variable increases the other decreases. As we have a slope for direction, we report absolute value of *τ*. A *τ* value of |0.2| to |0.5| indicates a moderate trend and $$ > |0.5|$$ indicates a strong trend.

## Results

### Annual area-normalized magnitude in Florida lakes

**Figure 3:**
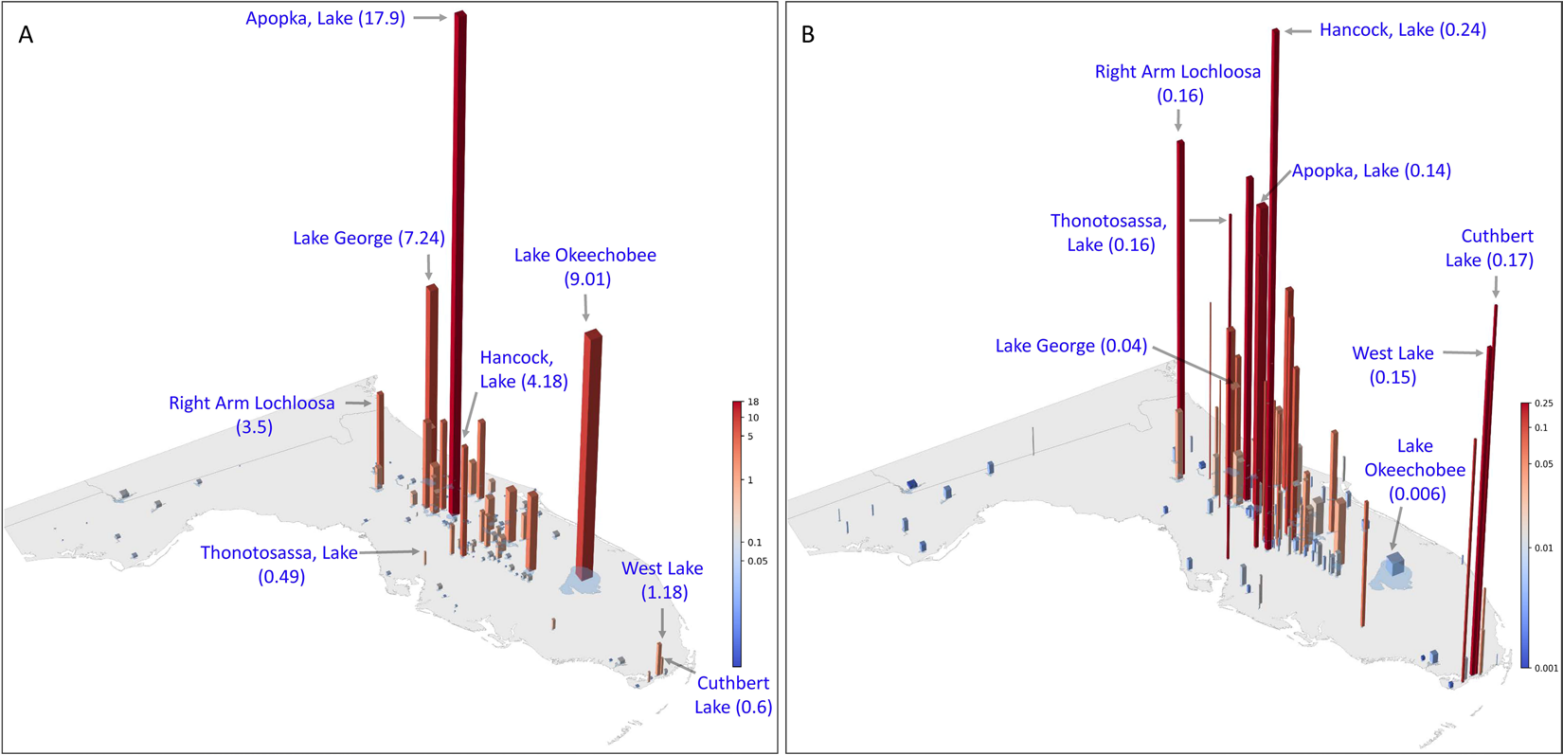
(**A**). Algal bloom magnitude in Florida lakes in 2011 before normalization and (**B)** after normalization by lake surface area. Area-normalized magnitude (km^−2^) of selected lakes provided as part of bar labels in parenthesis. Bar height and color are proportional to annual bloom magnitude (in **A**) and annual area-normalized magnitude (in **B**). The width of the bars is proportional to the lake surface area. Note that the color bars are log-scaled.

CyanoHAB magnitude is calculated as an annual mean of 7- or 14-day maximum accumulation of CI in the lakes in Florida over the observation period. Therefore, large lakes are more likely to have a higher accumulation of biomass as compared to lakes with smaller surface area (Fig. 3). Normalization of magnitude by lake surface area removes the influence of lake size from the metric and allows comparison of cyanobacteria area-normalized magnitude among lakes of different sizes. Results from 2011 are shown in Fig. 3, which highlights the impact of normalization of annual bloom magnitude using the lake surface area. Before normalization, Lake Apopka, Lake Okeechobee, Lake George, Hancock Lake, and Right Arm Lochloosa Lake ranked 1^st^ to 5^th^ respectively in 2011. Lake Apopka, which has the fourth largest surface area, but year-round blooms, ranked first, whereas Lake Okeechobee, with the largest surface area but less frequent blooms, ranked second in terms of cyanoHAB magnitude in 2011 (Fig. 3A). When the mean annual biomass estimates were normalized by lake surface area, Hancock Lake, Cuthbert Lake, Thonotosassa Lake, Right Arm Lochloosa, and West Lake ranked in the top five positions respectively (Fig. 3B). These results highlight that: 1) in 2011, while Lake Okeechobee had an intense bloom that covered only a portion of the lake, most of Hancock Lake was impacted; and 2) the average area of Hancock Lake was more severely affected by cyanobacteria than the average area in Lake Okeechobee.

**Figure 4:**
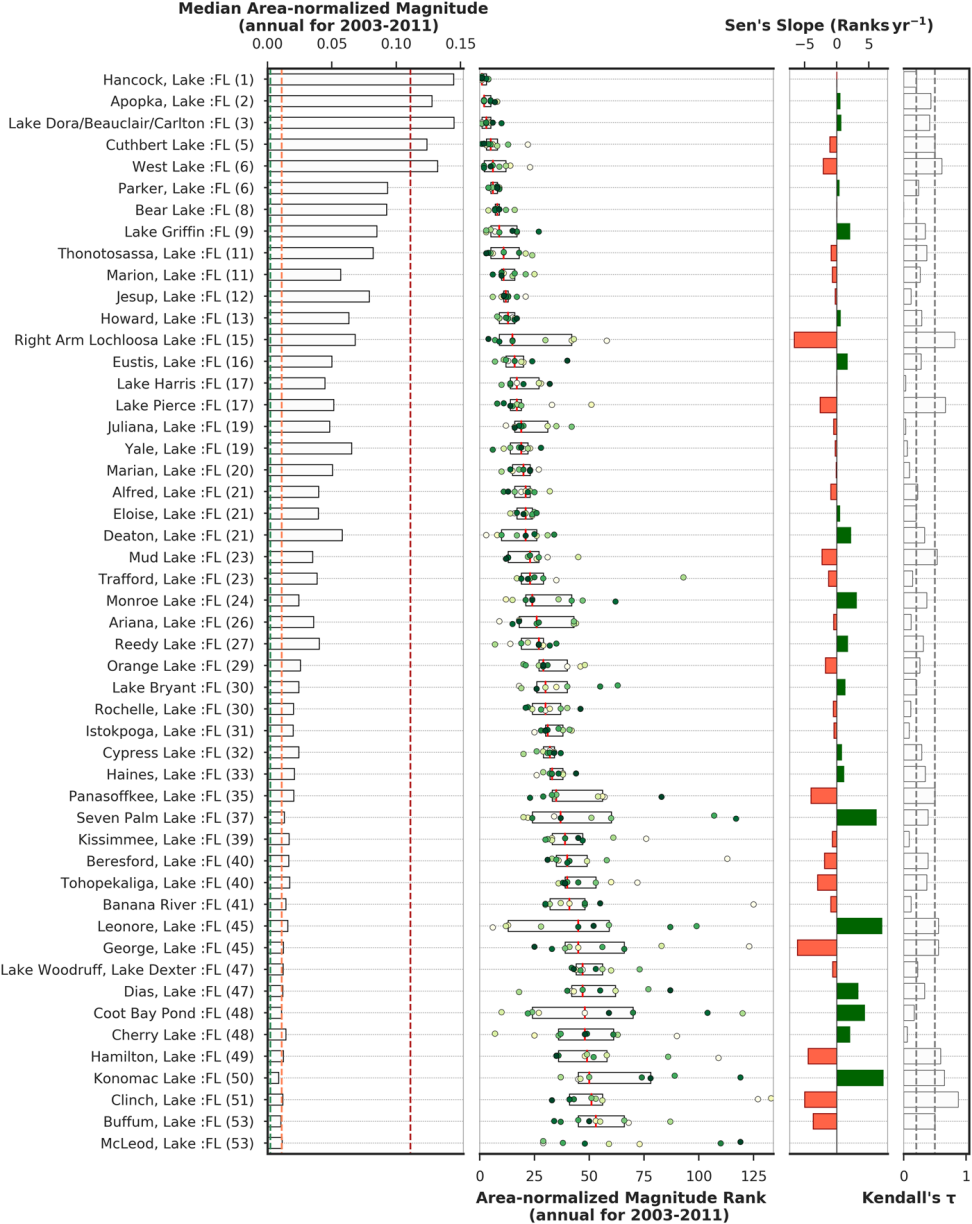
First panel: Annual area-normalized magnitude (km^−2^) in the top 50 Florida lakes. Green, orange, and red dotted lines represent equivalent WHO thresholds of 20,000, 100,000, and 1,000,000 cells mL^−1^ limits; second panel: the inter-quartile range of area-normalized magnitude ranks in top-ranked Florida lakes over 2003–2011 ordered by their median rank over the 9 year period. Median values or ranks are highlighted in red vertical lines inside the box. Annual area-normalized magnitude rank data points are overlaid on inter-quartile boxes to highlight the variation, where the color of the scatter points indicate data year (tan: start year and deep green: end year). Third panel: green/red bar plot shows Sen’s slope (trends in rank change) during 2003–2011. Green/red color represents positive/negative trend meaning area-normalized magnitude for a lake is decreasing/increasing over time. Fourth panel: bars show Kendall’s *τ* (absolute values) representing consistency in rank-change trend over time. Dotted lines in Kendall’s *τ* plot mark the *τ* values at 0.2 and 0.5.

Area-normalized magnitude rankings for all lakes during the study period were analyzed to identify those lakes with the most severe cyanobacteria blooms needing attention (Fig. 4). Lakes are ordered by their median rank from 2003–2011 in ascending order. Hancock Lake, Lake Apopka, Lake Dora/Beauclair/Carlton, Cuthbert Lake, and West Lake are the top five lakes for annual area-normalized bloom magnitude in Florida (Fig. 4, Table 1). The three top-ranked Florida lakes exhibited little variation over time (Hancock Lake, Lake Apopka, and Lake Dora/Beauclair/Carlton) (Fig. 4). Obviously, for a change in rank to occur one lake changes to a lower rank, another lake must move to a higher rank. The changes are not evenly distributed. Six lakes showed large declines: Right Arm Lochloosa and Lake George declined at ~6 ranks yr^−1^, and Clinch, Hamilton, Panasoffkee, and Buffum lakes declined at 3 to 5 ranks yr^−1^. Of these lakes, the decline was highly consistent (*τ* > 0.5) for Lochloosa, Panasoffkee, George, and Buffum, and moderately consistent (*τ* = 0.3–0.5) for the others. In contrast, only three lakes (Seven Palms, Leonore, Konomac) had consistent (*τ* > 0.3) and large increases in rank (better) changing at +6–7 ranks yr^−1^. Overall, area-normalized magnitude improved in Dias Lake, Monroe Lake, Deaton Lake, and Lake Griffin, which resulted in their lake ranks increasing at the rate of ~ + 2–3 ranks yr^−1^. Several of the lakes at the southern tip of Florida (e.g., Cuthbert, West), while in the Everglades, are actually estuarine with salinity influenced by Florida Bay. These are noted by asterisks (*) in the table.

**Table 1:** Summary of median annual bloom magnitude, median annual area-normalized magnitude and their ranks of top 50 lakes in Florida (2003–2011). +Lake area represented by MERIS pixels. * Indicates estuarine lake at the southern tip of Florida.

Lake Name	Surface Area^+^ (km^2^)	State	Median Bloom Magnitude (dl)	Median Area-normalized Magnitude (km^−2^)	Median Rank	Sen’s Slope (Ranks yr^−1^)	Kendall’s *τ*
Hancock, Lake	17.01	FL	2.457	0.144	1	0.00	−0.20
Apopka, Lake	121.5	FL	15.497	0.128	2	0.46	0.43
Lake Dora/Beauclair/Carlton	21.24	FL	3.077	0.145	3	0.65	0.41
Cuthbert Lake*	3.42	FL	0.423	0.124	5	−1.10	−0.50
West Lake*	7.47	FL	0.987	0.132	6	−2.13	−0.61
Parker, Lake	7.65	FL	0.712	0.093	6	0.35	0.24
Bear Lake*	3.15	FL	0.291	0.092	8	0.00	0.00
Lake Griffin	38.88	FL	3.297	0.085	9	2.00	0.34
Thonotosassa, Lake	2.97	FL	0.243	0.082	11	−0.88	−0.37
Marion, Lake	10.71	FL	0.607	0.057	11	−0.70	−0.26
Jesup, Lake	29.61	FL	2.338	0.079	12	−0.27	−0.11
Howard, Lake	2.16	FL	0.137	0.063	13	0.55	0.29
Right Arm Lochloosa Lake	21.51	FL	1.461	0.068	15	−6.63	−0.82
Eustis, Lake	29.79	FL	1.485	0.050	16	1.62	0.28
Lake Harris	71.1	FL	3.166	0.045	17	0.00	0.03
Lake Pierce	13.77	FL	0.707	0.051	17	−2.58	−0.67
Juliana, Lake	3.51	FL	0.169	0.048	19	−0.50	−0.03
Yale, Lake	14.94	FL	0.974	0.065	19	−0.26	−0.06
Marian, Lake	18.27	FL	0.920	0.050	20	−0.08	−0.09
Alfred, Lake	2.43	FL	0.096	0.040	21	−0.95	−0.22
Eloise, Lake	4.14	FL	0.163	0.039	21	0.45	0.20
Deaton, Lake	1.8	FL	0.104	0.058	21	2.13	0.33
Mud Lake	1.71	FL	0.060	0.035	23	−2.31	−0.54
Trafford, Lake	5.49	FL	0.211	0.038	23	−1.27	−0.14
Monroe Lake*	2.7	FL	0.065	0.024	24	3.00	0.37
Ariana, Lake	3.69	FL	0.132	0.036	26	−0.50	−0.11
Reedy Lake	13.41	FL	0.538	0.040	27	1.65	0.31
Orange Lake	23.58	FL	0.602	0.026	29	−1.79	−0.25
Lake Bryant	3.78	FL	0.091	0.024	30	1.27	0.20
Rochelle, Lake	1.98	FL	0.040	0.020	30	−0.55	−0.11
Istokpoga, Lake	90.99	FL	1.797	0.020	31	−0.45	−0.08
Cypress Lake	11.61	FL	0.282	0.024	32	0.73	0.29
Haines, Lake	2.52	FL	0.052	0.021	33	1.08	0.34
Panasoffkee, Lake	9.99	FL	0.204	0.020	35	−4.00	−0.50
Seven Palm Lake*	5.04	FL	0.065	0.013	37	6.08	0.39
Kissimmee, Lake	118.8	FL	1.969	0.017	39	−0.71	−0.08
Beresford, Lake	2.7	FL	0.044	0.016	40	−1.90	−0.39
Tohopekaliga, Lake	64.71	FL	1.113	0.017	40	−3.00	−0.37
Banana River	3.24	FL	0.046	0.014	41	−0.95	−0.11
Leonore, Lake	1.35	FL	0.021	0.016	45	7.00	0.56
George, Lake	172.71	FL	2.092	0.012	45	−6.13	−0.56
Lake Woodruff, Lake Dexter	17.37	FL	0.208	0.012	47	−0.63	−0.22
Dias, Lake	2.52	FL	0.029	0.012	47	3.25	0.33
Coot Bay Pond*	4.14	FL	0.044	0.011	48	4.29	0.17
Cherry Lake	1.62	FL	0.023	0.014	48	2.00	0.06
Hamilton, Lake	8.1	FL	0.100	0.012	49	−4.47	−0.59
Konomac Lake	4.05	FL	0.035	0.009	50	7.20	0.65
Clinch, Lake	4.14	FL	0.049	0.012	51	−5.00	−0.87
Buffum, Lake	4.95	FL	0.051	0.010	53	−3.67	−0.50
McLeod, Lake	1.53	FL	0.017	0.011	53.5	6.45	0.40

**Figure 5:**
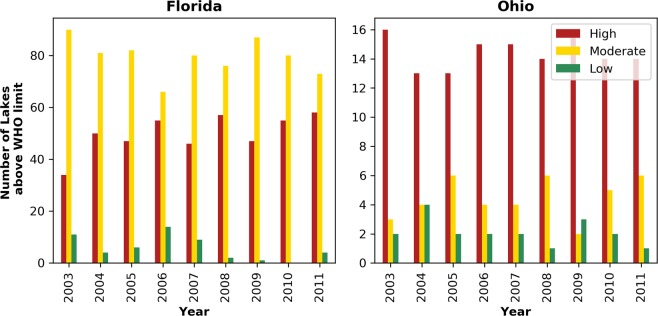
The number of lakes in Florida and Ohio classified as high, moderate, and low bloom categories based on recreational WHO cyanobacterial cell density limits.

In order to infer potential exposure risk of cyanoHABs in Florida lakes, we determined the number of lakes where cyanoHAB abundance exceeded the specific WHO risk thresholds of Low, Moderate, High, and V.High levels. A recreational Low WHO limit indicates lakes that are unlikely to have a management concern^48^. Out of 135 lakes, 34–58 (range represents the number of lakes in a specific year) lakes were assigned to the High category with area-normalized magnitude over the study period (Fig. 5A). 2011 witnessed the maximum number of lakes in the high category (n = 58, ~43% of all lakes in Florida). Similarly, the area-normalized magnitude for 66–90 lakes were in the Moderate category and 1–11 lakes were in the Low category. In 2010, all lakes were in Moderate and High categories. Area-normalized magnitude fell into the V.High range in 10 lakes (Right Arm Lochloosa Lake, Bear Lake, Parker Lake, Apopka Lake, Thonotosassa Lake, West Lake, Hancock Lake, Cuthbert Lake, Lake Griffin, and Lake Dora/Beauclair/Carlton, not in order) over the study period. In 2008 and 2011, eight (excluding Lake Griffin and Thonotosassa Lake) and nine (excluding Lake Griffin) out of those 10 lakes were categorized as V.High.

Without normalization by lake surface area, median magnitude data in Florida looked completely skewed (min = 0.007 CI, max = 15.49 CI, median = 0.05 CI), where about 90% of lakes were below annual bloom magnitude of one CI. The top 10% (13) having the highest median bloom magnitude had bloom magnitudes between 1.11–15.5 CI. Of these, six are also in the top 10% of the area-normalized magnitude (Apopka, Griffin, Dora/Beauclair/Carlton, Hancock, and Jesup). Some of these lakes with large bloom magnitude, e.g., Istokpoga, Kissimmee, and Tohopekaliga, ranked in the 2^nd^ quartile (27 and below) when area-averaged.

### Seasonal area-normalized magnitude in Ohio lakes

**Figure 6:**
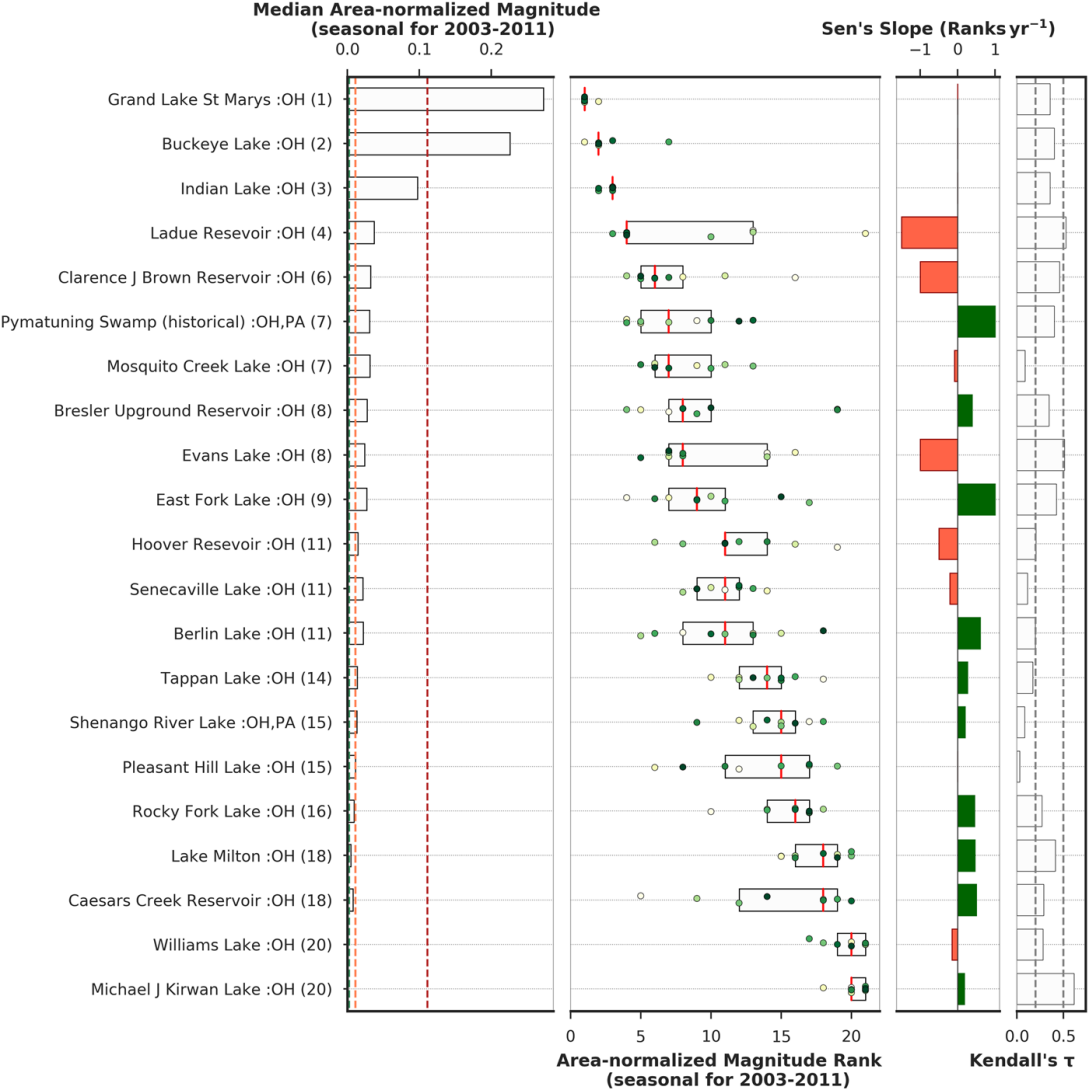
First panel: Seasonal area-normalized magnitude (km^−2^) in Ohio lakes. Green, orange, and red dotted lines represent equivalent WHO thresholds of 20,000, 100,000, and 1,000,000 cells mL^−1^ limits; second panel: the inter-quartile range of area-normalized magnitude ranks in top-ranked Ohio lakes over 2003–2011 ordered by their median rank over the 9 year period. Median values or ranks are highlighted in red vertical lines inside the box. Annual area-normalized magnitude rank data points are overlaid on inter-quartile boxes to highlight the variation, where the color of the scatter points indicate data year (tan: start year and deep green: end year). Third panel: green/red bar plot shows Sen’s slope (trends in rank change) during 2003–2011. Green/red color represents positive/negative trend meaning area-normalized magnitude for a lake is decreasing/increasing over time. Fourth panel: bars show Kendall’s *τ* (absolute values) representing consistency in rank-change trend over time. Dotted lines in Kendall’s *τ* plot mark the *τ* values at 0.2 and 0.5.

**Figure 7:**
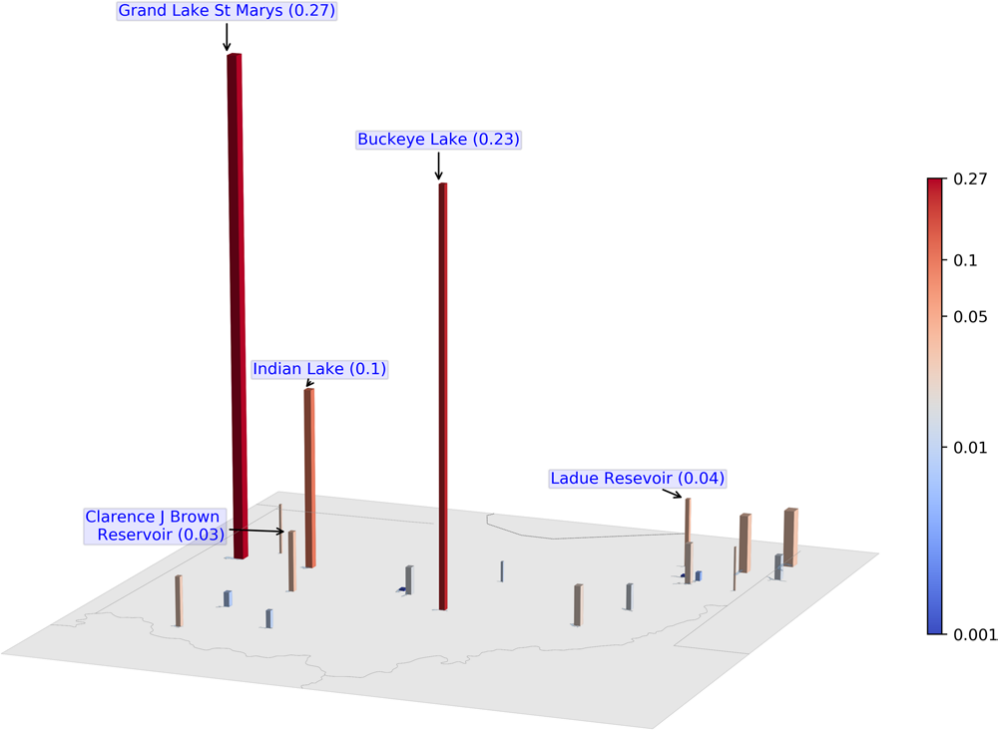
Bar plot showing median area-normalized magnitude (km^−2^) in Ohio lakes during the recreational season over the study period (2003–2011). Width of bars is proportional to lake surface area, height and color of the bars are proportional to the median annual area-normalized magnitude (km^−2^). Median values of the top five lakes are provided as part of bar labels inside the parenthesis.

CyanoHAB magnitude analyses in Ohio lakes during the recreational season (May 1^st^ to Oct 31^st^) showed that Grand Lake St. Marys (Rank 1, IQR = 0), Buckeye Lake (Rank 2, IQR = 0.25), and Indian Lake (Rank 3, IQR = 0.25) are the top three lakes by median seasonal area-normalized magnitude rank in Ohio from 2003–2011 (Figs. 6–7, Table 2). These top three lakes maintained the ranks consistently and were in WHO High category with area-normalized magnitude > 0.011 confirming severe CyanoHAB issue over time. Unlike the case for Florida, the variance in rank change for Ohio lakes during the study period is negligible, indicating that lakes maintained their area-normalized magnitude ranks consistently every year. However, there were substantial differences in cyanoHAB magnitude among different Ohio lakes. For instance, the median area-normalized magnitude in Grand Lake St. Marys (0.27 km^−2^) was ~12 times higher than 50% of the lakes in Ohio (Table 2). Out of 21 lakes in Ohio, there are three lakes – Ladue Reservoir, Clarence J. Brown Reservoir, and Evans Lake, where the relative rank decreased, or the area-normalized magnitude deteriorated over time (slope = ~1–1.5 ranks yr^−1^). These lakes showed relative decline against other lakes that showed relative improvement, especially East Fork Lake, Pymatuning reservoir, Lake Milton, Bresler Upground Reservoir, and Michael J Kirwan Lake.

**Table 2:** Summary of median seasonal bloom magnitude, median seasonal area-normalized magnitude and their ranks and statistics (Sen’s Slope and Kendall’s *τ*) of lakes in Ohio (2003–2011). Seasonal data (May 1^st^-Oct 31^st^). ^+^Lake area represented by MERIS FR pixels.

Lake Name	Surface Area^+^ (km^2^)	State	Median Bloom Magnitude (dl)	Median Area-normalized Magnitude (km^−2^)	Median Rank	Sen’s Slope (Ranks yr^−1^)	Kendall’s *τ*
Grand Lake St. Marys	47.97	OH	13.07	0.272	1	0	−0.35
Buckeye Lake	9.27	OH	2.091	0.226	2	0	0.4
Indian Lake	16.29	OH	1.591	0.098	3	0	−0.36
Ladue Reservoir	4.77	OH	0.177	0.037	4	−1.5	−0.53
Clarence J Brown Reservoir	7.38	OH	0.24	0.032	6	−1	−0.46
Pymatuning Reservoir	53.1	OH, PA	1.636	0.031	7	1	0.4
Mosquito Creek Lake	27	OH	0.846	0.031	7	−0.08	−0.09
Bresler Upground Reservoir	2.25	OH	0.061	0.027	8	0.39	0.34
Evans Lake	1.98	OH	0.047	0.024	8	−1	−0.51
East Fork Lake	6.57	OH	0.176	0.027	9	1	0.42
Hoover Reservoir	9.72	OH	0.145	0.015	11	−0.5	−0.2
Senecaville Lake	11.61	OH	0.248	0.021	11	−0.21	−0.11
Berlin Lake	8.91	OH	0.197	0.022	11	0.61	0.2
Tappan Lake	6.75	OH	0.092	0.014	14	0.27	0.17
Shenango River Lake	11.07	OH, PA	0.146	0.013	15	0.2	0.08
Pleasant Hill Lake	2.43	OH	0.027	0.011	15	0	−0.03
Rocky Fork Lake	6.12	OH	0.057	0.009	16	0.45	0.27
Lake Milton	5.94	OH	0.028	0.005	18	0.46	0.41
Caesars Creek Reservoir	9.09	OH	0.071	0.008	18	0.5	0.29
Williams Lake	9.72	OH	0.008	0.001	20	−0.15	−0.28
Michael J Kirwan Lake	7.92	OH	0.007	0.001	20	0.18	0.61

Based on the WHO recreational thresholds, 13 to 16 lakes (~62–76%) had an area-normalized magnitude in the High category (Fig. 5B). Years 2003 and 2009 had the maximum number of lakes (n = 16) in the High magnitude category and years 2004 and 2005 had the minimum number of lakes (n = 13) in the High category. Similarly, 2–6 (~10–29%) and 1–4(~5–14%) lakes were under Moderate and Low categories, respectively. Area-normalized magnitude in Grand Lake St. Marys stayed in the V.High category throughout the study period. Similarly, the area-normalized magnitude in Buckeye Lake fell in the V.High category during 2003–2007 and in 2011. Bloom magnitude in Indian Lake reached the V.High threshold only in 2007. Median bloom magnitude data before normalization clearly highlighted four lakes: Grand Lake St. Marys (13.07 CI), Buckeye Lake (2.09 CI), Pymatuning Reservoir (1.64 CI), and Indian Lake (1.59 CI), where bloom magnitudes were one order of magnitude higher than the rest of the lakes.

### Comparison of area-normalized magnitude in Florida and Ohio lakes

In order to compare Florida and Ohio, the magnitudes from the two states were combined and lakes were then ranked. In the combined dataset, the seasonal area-normalized magnitude estimation was limited to a time period of May 1^st^–Oct 31^st^ for each year to exclude the snow/ice-covered winter months and to include area-normalized magnitude status during the typical cyanoHAB season. In addition, a comparison between the two states was only performed from 2008–2011 to avoid any positive bias in the MERIS FR data in Ohio relative to Florida prior to the year 2008.

**Figure 8:**
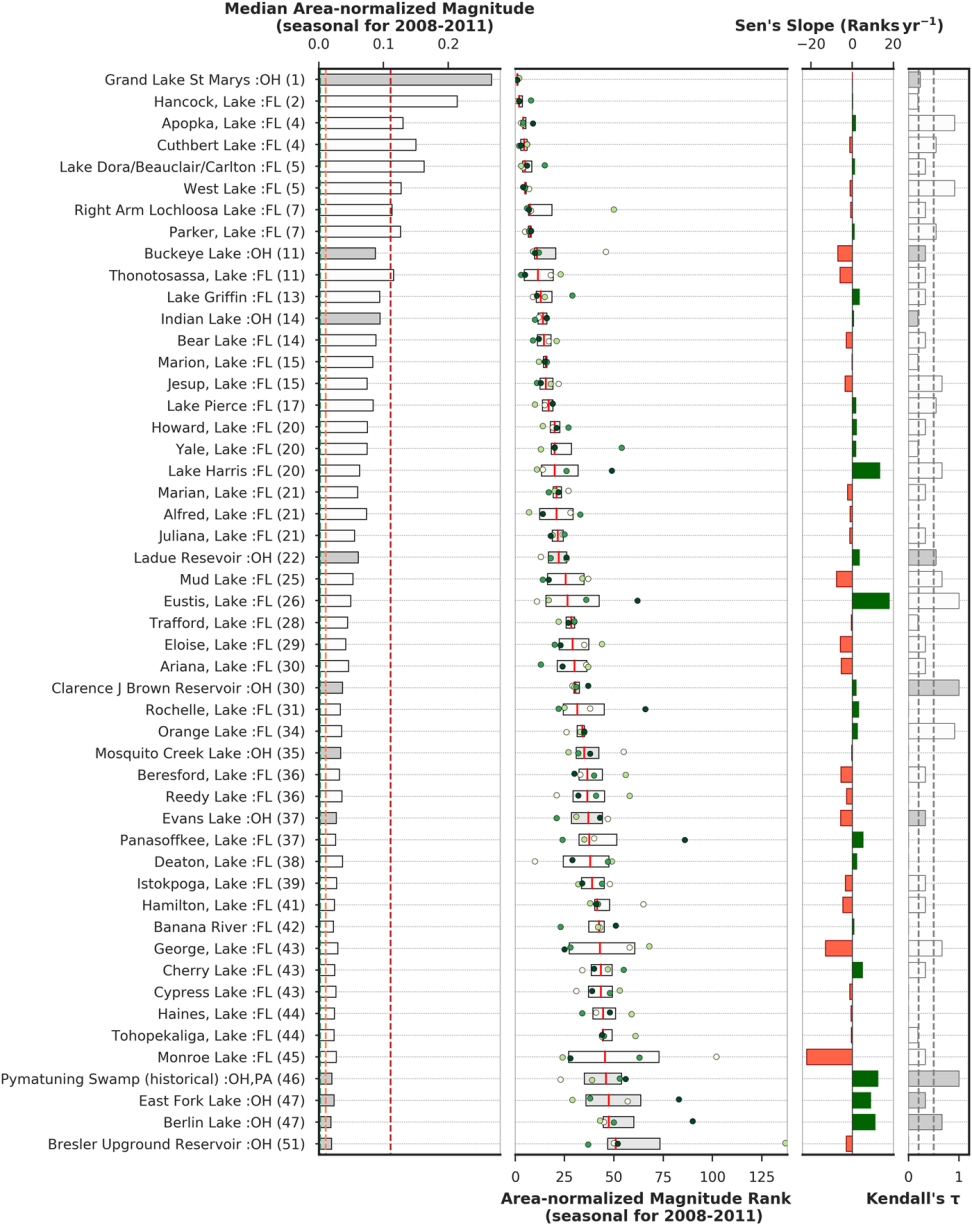
First panel: Seasonal area-normalized magnitude (km^−2^) in the top 50 Florida and Ohio lakes. Green, orange, and red dotted lines represent the equivalent WHO thresholds of 20,000, 100,000, and 1,000,000 cells mL^−1^ limits. Lakes with white and shaded bars are located in Florida and Ohio, respectively; second panel: the inter-quartile range of area-normalized magnitude ranks in top-ranked Florida lakes over 2008–2011 ordered by their median rank over the 4 year period. Median values or ranks are highlighted in red vertical lines inside the box. Annual area-normalized magnitude rank data points are overlaid on inter-quartile boxes to highlight the variation, where the color of the scatter points indicate data year (tan: start year and deep green: end year). Third panel: green/red bar plot shows trends in rank change during 2008–2011. Green/red color represents positive/negative trend meaning area-normalized magnitude for a lake is decreasing/increasing over time. Fourth panel: bars show Kendall’s *τ* (absolute values) representing consistency in rank-change trend over time. Dotted lines in Kendall’s *τ* plot mark the *τ* values at 0.2 and 0.5.

**Figure 9:**
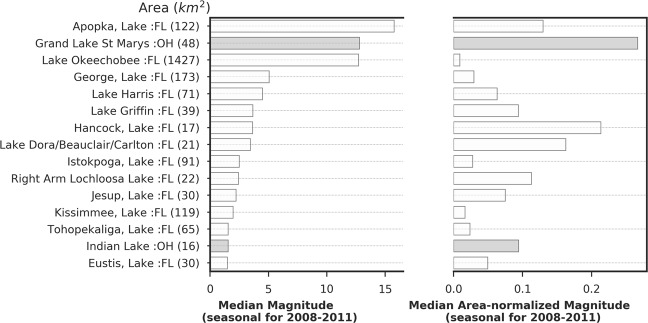
Left panel: Median seasonal bloom magnitude in 15 Florida and Ohio lakes over the study period (2008–2011) ordered by their values. Lake labels include the state name the lake is associated with and the number inside brackets represents the median area-normalized magnitude rank over the same study period. Right panel: median area-normalized magnitude for the same lakes during the same study period provided for comparison. Gray-colored bars represent lakes from Ohio.

Among all lakes from the two states, Grand Lake St. Marys (OH), Hancock Lake (FL), Apopka Lake (FL), Cuthbert Lake (FL), Lake Dora/Beauclair/Carlton (FL), West Lake (FL), Right Arm Lochloosa Lake (FL), Parker Lake (FL), Buckeye Lake (OH), and Thonotosassa Lake (FL) are among the top ten lakes based on median area-normalized magnitude rank as observed from 2008 to 2011 (Fig. 8, Table 3). Based on seasonal magnitude (not normalized), Apopka Lake in Florida (15.75 CI), Grand Lake St. Marys in Ohio (12.78 CI), and Lake Okeechobee in Florida (12.71 CI) are top three lakes in descending order (Fig. 9), reflecting the large magnitude blooms that occurred in these large lakes.

**Table 3:** Summary of median seasonal bloom magnitude, median seasonal area-normalized magnitude and their ranks and statistics (Sen’s Slope and Kendall’s *τ*) of lakes in Florida and Ohio (2008–2011). ^+^Surface area determined by MERIS FR pixels within the lake polygon. * Indicates estuarine lake at the southern tip of Florida.

Lake Name	Surface Area (km^2^)^+^	State	Median Bloom Magnitude (dl)	Median Area-normalized Magnitude (km^−2^)	Median Rank	Sen’s Slope (Ranks yr^−1^)	Kendall’s *τ*
Grand Lake St. Marys	47.97	OH	12.788	0.267	1.00	0.00	−0.24
Hancock, Lake	17.01	FL	3.630	0.213	2.00	0.25	0.18
Apopka, Lake	121.50	FL	15.754	0.130	4.00	1.50	0.91
Cuthbert Lake*	3.42	FL	0.513	0.150	4.50	−1.25	−0.55
Lake Dora/Beauclair/Carlton	21.24	FL	3.452	0.163	5.00	1.08	0.33
West Lake*	7.47	FL	0.950	0.127	5.00	−1.00	−0.91
Right Arm Lochloosa Lake	21.51	FL	2.424	0.113	7.50	−0.67	−0.33
Parker, Lake	7.65	FL	0.963	0.126	7.50	1.00	0.55
Buckeye Lake	9.27	OH	0.809	0.087	11.00	−7.00	−0.33
Thonotosassa, Lake	2.97	FL	0.342	0.115	11.50	−5.92	−0.33
Lake Griffin	38.88	FL	3.649	0.094	13.00	3.33	0.33
Indian Lake	16.29	OH	1.535	0.094	14.00	0.67	0.18
Bear Lake*	3.15	FL	0.278	0.088	14.50	−2.83	−0.33
Jesup, Lake	29.61	FL	2.212	0.075	15.50	−3.50	−0.67
Marion, Lake	10.71	FL	0.893	0.083	15.50	−0.17	−0.18
Lake Pierce	13.77	FL	1.155	0.084	17.00	1.67	0.55
Lake Harris	71.10	FL	4.481	0.063	20.00	13.33	0.67
Yale, Lake	14.94	FL	1.114	0.075	20.00	1.75	0.18
Howard, Lake	2.16	FL	0.162	0.075	20.00	2.08	0.33
Marian, Lake	18.27	FL	1.093	0.060	21.00	−2.33	−0.33
Alfred, Lake	2.43	FL	0.179	0.073	21.00	−1.08	0.00
Juliana, Lake	3.51	FL	0.194	0.055	21.50	−1.25	−0.33
Ladue Resevoir	4.77	OH	0.291	0.061	22.00	3.42	0.55
Mud Lake	1.71	FL	0.090	0.053	25.50	−7.58	−0.67
Eustis, Lake	29.79	FL	1.475	0.050	26.50	18.00	1.00
Trafford, Lake	5.49	FL	0.244	0.044	28.50	−0.50	−0.18
Eloise, Lake	4.14	FL	0.172	0.042	29.00	−5.75	−0.33
Ariana, Lake	3.69	FL	0.169	0.046	30.00	−5.25	−0.33
Clarence J Brown Reservoir	7.38	OH	0.270	0.037	30.50	1.83	1.00
Rochelle, Lake	1.98	FL	0.066	0.033	31.50	3.17	0.00
Orange Lake	23.58	FL	0.834	0.035	34.00	2.50	0.91
Mosquito Creek Lake	27.00	OH	0.912	0.034	35.00	−0.33	0.00
Beresford, Lake	2.70	FL	0.086	0.032	36.50	−5.50	−0.33
Reedy Lake	13.41	FL	0.479	0.036	36.50	−2.67	0.00
Evans Lake	1.98	OH	0.053	0.027	37.00	−5.67	−0.33
Panasoffkee, Lake	9.99	FL	0.259	0.026	37.50	5.17	0.00
Deaton, Lake	1.80	FL	0.066	0.036	38.00	2.17	0.00
Istokpoga, Lake	90.99	FL	2.493	0.027	39.00	−3.33	−0.33
Hamilton, Lake	8.10	FL	0.193	0.024	41.50	−4.50	−0.33
Banana River	3.24	FL	0.074	0.023	42.50	0.83	0.00
George, Lake	172.71	FL	5.049	0.029	43.00	−13.00	−0.67
Cypress Lake	11.61	FL	0.306	0.026	43.50	−1.17	0.00
Cherry Lake	1.62	FL	0.040	0.025	43.50	5.00	0.33
Tohopekaliga, Lake	64.71	FL	1.536	0.024	44.50	−0.50	−0.18
Haines, Lake	2.52	FL	0.060	0.024	44.50	−0.58	0.00
Monroe Lake*	2.70	FL	0.073	0.027	45.50	−22.08	−0.33
Pymatuning Reservoir	53.10	OH, PA	1.056	0.020	46.00	12.50	1.00
Berlin Lake	8.91	OH	0.168	0.019	47.50	11.00	0.67
East Fork Lake	6.57	OH	0.154	0.023	47.50	8.83	0.33
Bresler Upground Reservoir	2.25	OH	0.044	0.019	51.00	−2.92	0.00

## Discussion

Evaluating severity each year and comparing lakes provides a potentially important resource for managers, including under such regulatory frameworks as the European Union Water Framework Directive^52^ and the U.S. Clean Water Act^53,54^. In Lake Erie, an assessment of magnitude (over a 30-day period) from satellite proved essential to the development of nutrient target strategies^55^, and has also led to an annual forecast of bloom severity^45^. In comparison to satellite, traditional routine monitoring is difficult and expensive. Exceptionally strong programs such as Ohio EPA’s drinking water program may collect a sample each week in a water body (although for toxin only)^56^. More common for a water quality monitoring program is monthly or quarterly sampling of water quality (including Chl-*a*), such as seen in Florida’s several monitoring programs^57,58^.

The results presented here capture the relative severity of cyanobacterial blooms observed in state monitoring programs. In Ohio, the concentration of microcystin toxins is the most common water quality measurement. Three of the top four lakes (Grand Lake St. Marys, Buckeye, and LaDue) consistently reported the highest microcystin concentrations of the observed lakes when Ohio EPA started sampling in 2010, and these were well above the WHO recreational risk levels (10 µg L^−1^)^15^. These lakes are also currently listed as impaired due to algae and associated microcystin^56^. The fourth, Indian Lake, does not have routine sampling.

**Figure 10:**
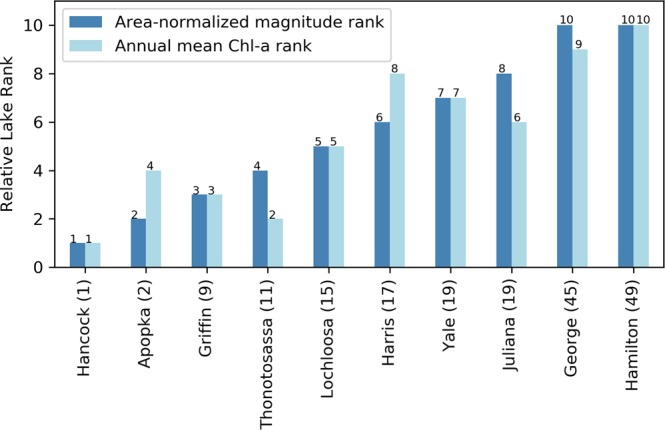
Relative comparison of lake ranks calculated from the annual area-normalized magnitude and measured annual mean Chl-*a* concentration. Numbers associated with the lake names in the x-axis tick label represent the median lake rank as in Table 1.

In Florida, we verified the ranking using field Chl-*a* data for lakes found in both the Florida Water Atlas^58^ and in the top 50 lakes from our satellite-based observations (as in Table 1). Similar to annual area-normalized magnitude estimation, we calculated annual mean Chl-*a* concentration for the study years 2003–2011 by taking the mean of monthly mean Chl-*a* concentrations for all samples from a lake available in the database. To match area-normalized magnitude, we calculated the median of annual mean Chl-*a* concentrations over the study period. We further ranked the ten lakes based on median area-normalized magnitude and median of annual mean Chl-*a* concentration over nine-year study period (Fig. 10), and found similar results across these lakes from Lake Hancock (1 in both) to Hamilton Lake (rank 10 among this set of lakes, and 49 in the larger satellite dataset).

Two studies with satellite-based methods examined Ohio and Florida lakes, and had similar conclusions about the most impacted lakes as found here, although they use different methods. Gorham *et al*.^27^ used 10 years of MERIS data (2002–2011) to estimate PC with a semi-analytical model^25^. Lakes were evaluated using the maximum PC concentration for the year at each pixel in each lake. This approach would rank a lake having a single day of high concentration as more severe than a lake with a slightly less severe long-duration bloom. Regardless, this approach also put Grand Lake St. Marys, Buckeye Lake, Indian Lake, and Seneca Lake as the top four, matching our result.

Clark *et al*.^15^ focused on bloom frequency. They calculated cyanoHAB frequency for Ohio and Florida lakes as the fraction of total pixel observations where cyanoHAB abundance exceeded the WHO’s threshold of 100,000 cells mL^−1^, and then ranked those lakes by the overall frequency during 2008–2011 study period. They concluded that Lake Apopka in Florida (cyanoHAB frequency = 99.1%) and Grand Lake St. Marys in Ohio (cyanoHAB frequency = 83.1%) had the highest cyanoHAB frequency during 2008–2011. Their top ten lakes from Florida based on cyanoHAB bloom frequency^15^ (in order: Apopka (1), Pierce, Dora, Marion, Howard, Parker, Hancock, Harris, Jesup, and Juliana (10)) are in the top 18 lakes in our study (Fig. 4). As expected, a comparison of Florida lake ranks based on the frequency and area-normalized magnitude highlights differences in information between the methods. A lake with a persistent moderate bloom would rank higher in frequency than in magnitude; an example is Lake Pierce (rank 2 in frequency, 9 in magnitude). A lake with short intense blooms would rank higher in magnitude than in frequency. An example is Hancock Lake, which has intense annual blooms (Chl-*a* of 300–500 mg m^−3^) that only last for a few months^58^, and ranks 1 in area-normalized magnitude and 7 in frequency. The difference between metrics can be more acute in large lakes, like Lake Okeechobee, which have blooms that are large in magnitude but do not cover most of the lake (Fig. 9). A single metric cannot highlight all aspects of cyanoHABs; complementary cyanoHAB metrics representing factors like bloom frequency, area, and area-normalized magnitude are needed.

Wind-driven mixing in the water column can add uncertainty to the cyanoHAB magnitude estimates for species with buoyancy regulation, such as *Microcystis aeruginosa* and *Aphanizomenon flos-aquae*. Satellite algorithms, in bloom conditions, detect cyanobacteria concentration only near the surface. Previous studies have reported that wind stress can increase vertical mixing of cyanobacteria cells and thereby reduce the ability of the ocean or water color sensor to detect the majority of the biomass^12^. Therefore, persistent high wind (>7.7 m s^−1^ as observed in Lake Erie)^11^, when combined with frequent cloud cover (such that only windy days during the bloom season are imaged) may occasionally lead to underestimation of cyanoHAB biomass or area-normalized magnitude. Cloud cover, sun glint, and the effectiveness of masking algorithms for other invalid pixels (e.g. mixed land and water at the shore, dry lake bed, algal mats, and vegetative areas) may add uncertainty to the satellite-based measurements. Cloud and glint impacts should be uniform through a region, but the other factors might bias specific lakes. Another source of uncertainty could come from the use of the CI-cyanobacteria cell count relationship when the analysis would be scaled up to the CONUS lakes. In Lunetta *et al*.^41^, a CI-cyanobacteria biomass relationship was demonstrated using field data collected from lakes in the US states across Ohio, Florida, and throughout New England. The same CI-cyano and cyanobacteria biomass relationship was revised and presented in Clark *et al*.^15^ with more meaningful error estimates (MAPE = 28.6%). Error in manual cell enumeration is inversely proportional to the number of colonies counted^48^. 20–30% error is expected and considered acceptable when at least 400 units or colonies are counted although Chorus and Bartram^48^ report that normally 20–40 colonies may be present in 100 mL of sampled water from the field. Therefore, even higher than 20–30% error, only from field cell density, cannot be ruled out. Similarly, variability in the spatial distribution of biomass in a bloom can add up to two orders of magnitude difference in biomass, as observed by^59^ in a cyanobacteria bloom in the Gulf of Finland. Spatiotemporal variability in biomass in a diurnal scale can add significant uncertainty as well^60^. Therefore, after considering errors from multiple sources, it is expected to have greater than 30% error or difference in satellite estimates, when compared with field measured cell density from a point source in a bloom event. Additionally, a CI-*cyano* to Chl-*a* relationship, established by Tomlinson *et al*.^16^ for Florida lakes, could be used in future studies, although the CI-*cyano* to Chl-*a* relationship may require additional examination before applying to all CONUS lakes.

Lake size presents a potential limitation on decisions based on area-normalized magnitude. One such example is Lake Okeechobee in Florida, which is the largest freshwater lake in Florida and the 9^th^ largest freshwater lake (by area) in the United States. Due to its size and societal importance (for water supply, tourism, and ecological impacts), cyanoHAB issues in this lake have been widely covered by the press and media, thereby creating cyanoHAB awareness at state and national levels. While Lake Okeechobee was ranked second in bloom magnitude (behind Apopka, a moderately large lake), based on the area-normalized magnitude, Lake Okeechobee was ranked 95^th^ among the Florida lakes. This is because the bloom area is simply a small percentage of surface area in such a large lake. Therefore, for larger lakes, annual or seasonal bloom magnitude numbers should be used for lake water management and decision making related to water quality. In contrast, area normalization highlights cyanoHABs in most of the smaller lakes such as Hancock Lake, Lake Dora/Beauclair/Carlton, and many others, which may not get enough attention due to their size, although they equally bear the potential of causing an adverse effect on health and the environment.

The proposed bloom magnitude metric provided a synoptic view in lakes by capturing spatiotemporal mean areal cyanobacteria biomass. Further normalization of bloom magnitude by lake surface area provided comparable and actionable information for water quality managers inside states or other jurisdictional boundaries. The relative ranking of lakes allows the MERIS record (2003–2011) to be utilized within a state or a region, as the MERIS FR temporal frequency is expected to be similar. Ranks and nonparametric statistics provide robust parameters that do not depend on the calibration accuracy and precise thresholds, as compared to the direct metrics like a bloom area, bloom frequency, or area-normalized magnitude. The rank-based metric has additional power of allowing the use of multiple satellites without introducing biases between the different satellite data sets. For example, OLCI on Sentinel 3A and 3B may not currently match the MERIS calibration as the OLCI calibration is still on-going as of this writing, but the ranking of the lakes would eliminate the systematic bias in the data due to differences in calibration coefficients. Therefore, area-normalized magnitude ranks estimated from OLCI should be consistent with those from MERIS in each season, allowing identification of those lakes that are changing in bloom magnitude. The area-normalized magnitude should be inspected (together with sample frequency) to confirm that there is not a systemic change in all the lakes in a region over the time period of interest. This problem is less likely to occur if many lakes are considered in the analysis, or if they have fundamentally different environmental characteristics. These sensors cannot resolve all lakes of interest in a state. Narrow lakes and some rivers are a particular problem. For those small or narrow water bodies, Sentinel-2 may provide a solution, but that requires more research, as some key bands (620 and 681 nm) are not on that sensor.

The method presented in this study captures an assessment of cyanoHAB magnitude, which is the cyanobacteria biomass for the year or season. Normalization of bloom magnitude by lake surface area let us compare the cyanoHAB magnitude across lakes with varying size. Our approach to rank the lakes by median area-normalized magnitude helped us to highlight the top lakes, which need immediate attention from water quality managers. Provided below are three advantages of the ranking:

1. This approach uses the power of ranked and non-parametric statistics in order to be able to use the MERIS time series (2003–2011) in a state or a localized region irrespective of bias in temporal coverage. However, if contemporaneous satellite data collection frequency is different between two areas, they cannot be compared side-by-side and lakes in those areas should be analyzed separately.
2. This approach would also allow the use of Sentinel-3 OLCI data along with MERIS time series even though the sensor calibration coefficients of OLCI are still being refined and may not match with MERIS. This would enable a comparison of cyanoHAB bloom magnitude derived from OLCI with the historic cyanoHAB magnitude derived from MERIS.
3. As our method also included WHO recreational thresholds, the same information may also be used for categorizing which lakes need pressing attention for cyanoHAB management. A specific threshold representing exposure risk can be set and lakes above that threshold may be identified as a priority during the observational period.

No one metric can completely represent the attributes of cyanoHAB severity of interest to water quality managers. Area-normalized magnitude can provide awareness of smaller lakes that have significant blooms. However, the area-normalized magnitude can equate a short-lived, large, intense bloom with a long-lived, moderate bloom in any sized lake. Therefore, the reader is encouraged to compare other metrics such as temporal frequency^15^ and bloom spatial extent^29^ to address related questions. These methods complement each other and can provide a more complete picture of cyanoHABs on a regional or national scale. The Cyanobacteria Assessment Network (CyAN) project^13^ provides the capability to scale this effort to CONUS fresh water lakes and water bodies. Our future work could focus on providing a comprehensive analysis of cyanoHAB magnitude in CONUS lakes and identifying lakes of concern.

## Data Availability

The satellite dataset used in the current study can be downloaded from https://oceancolor.gsfc.nasa.gov/ and datasets generated during this study are available from the corresponding author on request.
